# Depression and Anxiety Symptoms in Headache Disorders: An Observational, Cross-Sectional Study

**DOI:** 10.3390/neurolint16020026

**Published:** 2024-03-18

**Authors:** Leonidas Mantonakis, Ioanna Belesioti, Christina I. Deligianni, Vasilis Natsis, Euthimia Mitropoulou, Elina Kasioti, Maria Lypiridou, Dimos D. Mitsikostas

**Affiliations:** 1 First Psychiatry Department, Aeginition Hospital, Medical School, National & Kapodistrian University of Athens, V. Sofia’s Avenue 74, 11528 Athens, Greece; lmantonakis@gmail.com (L.M.); bill.natsis@hotmail.com (V.N.); 2 First Neurology Department, Aeginition Hospital, Medical School, National & Kapodistrian University of Athens, V. Sofia’s Avenue 74, 11528 Athens, Greece; belesioti.ioanna@gmail.com (I.B.); cdchristina@gmail.com (C.I.D.); goranbeauty@yahoo.gr (E.M.); elkasioti@hotmail.com (E.K.); marialypiridou@gmail.com (M.L.); 3 Neurology Department, Athens Naval Hospital, Deinokratous 70, 11521 Athens, Greece

**Keywords:** depression, anxiety, migraine, tension-type headache, medication overuse headache, cluster headache

## Abstract

Background: Headache disorders have been associated with anxiety and depressive disorders. The aim of this study was to assess symptoms of anxiety and depression in a large sample of individuals with different headache disorders (HDs) in order to determine whether their frequency differs by headache type. Methods: Consecutive individuals with headache attending a headache outpatient clinic were interviewed with the HAM-D and HAM-A, along with age, sex, and education matched non-headache individuals. Results: Individuals numbering 2673 with headache (females 71.2%) and 464 non-headache individuals (females 70.9%) were interviewed (with participation rates of 98.3% and 91.0%, respectively). Migraine was diagnosed in 49.7%, tension-type headache in 38%, cluster headache 5.2%, and medication overuse (MO) in 21.8%. Participants with HD scored more in HAM-A (OR = 4.741, CI95%: 3.855–5.831, *p* < 0.001) and HAM-D scales (OR = 2.319, CI95%: 1.892–2.842, *p* < 0.001) than non-headache individuals. Participants with chronic HDs (≥15 days with headache for ≥3 consecutive months; 52.5%) scored higher for both HAM-A (OR = 1.944, CI95%: 1.640–2.303, *p* < 0.001) and HAM-D (OR = 1.625, CI95%: 1.359–1.944, *p* < 0.001) than those with episodic HDs (33.1%), as did participants with MO vs. participants without MO (OR = 3.418, CI95%: 2.655–4.399, *p* < 0.001 for HAM-A, OR = 3.043, CI95%: 2.322–3.986, *p* < 0.001 for HAM-D). Female and low-educated participants scored higher on both scales. Conclusion: Because symptoms of anxiety and depression are substantial in people with HD, the treating physicians should look out for such symptoms and manage them appropriately.

## 1. Introduction

Headache and depressive disorders stood among the most prevalent health concerns, ranking within the top 15 causes of disability across 369 diseases and injuries globally in the year 2019 [1]. Notably, the burden of disorders has increased markedly over the last 20 years, within the young and productive age group of people, where both disorders are leading causes [1]; this underscores the pressing need for comprehensive understanding and effective management. Compounding this issue is the observation that headache and depressive disorders often coexist within the same individuals [2,3], multiplying the burden of both conditions and limiting the therapeutical outcome [4].

The undeniable association between headache and depressive disorders has prompted extensive research into the intricate mechanisms underlying this relationship. Genetic studies have shed light on a bidirectional influence [5], introducing complexity to our understanding of this comorbidity. This intricate interplay raises crucial questions about the mutual influence of these conditions and the underlying factors that contribute to their coexistence. Additionally, anxiety and depression have been identified as potential co-factors in the chronification of headaches, leading to suggestions of including anxiety and/or depression treatment as part of migraine management strategies [6].

This cross-sectional study seeks to contribute to the existing knowledge by estimating the prevalence of anxiety and depression-like symptoms in individuals with headache disorders (HDs). Thus, our aim was to explore potential associations with specific headache disorders, such as migraine, tension-type headache (TTH), and medication overuse (MO). To our knowledge there has been no controlled study investigating mood and anxiety symptoms in a sample of people with different HDs, although there are several studies that have focused on individual HDs, e.g., people with migraine, cluster headache, or TTH. Moreover, no study has so far investigated whether specific symptoms of anxiety or depression are more common in certain headache types, so we also aimed to compare the prevalence of gastrointestinal, sleep, and cardiovascular-related symptoms in different HDs. These symptoms are reported commonly by people with HD, people with additional anxiety and or depression in particular [6], and if present, require special management.

By elucidating these intricate connections, we aimed to provide valuable insights that can inform more targeted and effective interventions for individuals presenting with the dual challenges of headache and depressive disorders.

## 2. Materials and Methods

Consecutive individuals with HDs seeking medical treatment at the Aeginition Headache outpatient clinic, formed the participant pool for this cross-sectional study. Recruitment, interviewing, and clinical examination of participants were performed prospectively. The study encompassed a thorough examination of the main demographic features, clinical headache characteristics, and associated factors potentially contributing to the complexity of these disorders.

For each participant, detailed information on demographic features including age, sex, and educational background was systematically collected. Clinical headache features such as disease duration, frequency, and concurrent autonomic symptoms were carefully recorded during a comprehensive history-taking session. Provoking factors, including habits such as alcohol consumption, smoking, and coffee intake, were scrutinized to identify potential triggers.

Furthermore, a comprehensive assessment of coexisting somatic and mental disorders was integral to our investigation. This multifaceted exploration was facilitated through a structured questionnaire administered during the history-taking process. The questionnaire was designed to capture a spectrum of relevant details, ensuring a nuanced understanding of the participants’ health profiles. A crucial aspect of our methodology involved a meticulous physical and neurological examination for all participants.

Additionally, a specialized interview was conducted using the Hamilton Anxiety Rating Scale (HAM-A) and Hamilton Depression Rating Scale (HAM-D). The HAM-A was one of the first rating scales developed to measure the severity of anxiety symptoms and is still widely used today in both clinical and research settings. The administration time takes 10–15 min. The scale consists of 14 items, each defined by a series of symptoms measuring mental and somatic symptoms and scored on a scale of 0 (not present) to 4 (severe), with a total score range of 0–56, where <17 indicates mild severity, 18–24 mild to moderate severity, and 25–30 moderate to severe [7]. Similarly, the HAM-D, consisting of 17 items assessing both mental and somatic depressive symptoms, was utilized to gauge the severity of depression. The scale, ranges from 0 to 68, where 10–13 indicates mild depression; 14–17 mild to moderate; >17 moderate to severe depression [8,9]. The Cronbach’s alpha coefficient for the HAM-A and HAM-D varies depending on the specific version of the questionnaire and the population it is administered to. However, studies have reported Cronbach’s alpha values for the HAM-A and HAM-D ranging from around 0.70 to 0.90, indicating good to excellent internal consistency reliability.

Headache disorder diagnoses were conducted in accordance with the International Classification of Headache Disorders, 3rd edition (ICHD-3beta) [10]. Individuals with more than one HD were classified according to the most severe and disabling headache type. People with concomitant changes in cerebrospinal fluid pressure were excluded from the study population. All participants were stratified based on their education levels, categorized into three tiers (primary, secondary, and tertiary education). Outpatients who did not consent to participate in the study or did not agree to be interviewed for the HAM-A and HAM-D were excluded from the study.

We conducted a meticulous screening process using data from our hospital’s headache specialty clinic database to establish a diverse control group, encompassing both healthy individuals and patients without headache concerns. Control group participants underwent thorough evaluation procedures consistent with clinic standards, ensuring precise classification and eligibility for the study’s comparative analysis. Our paramount objective was to uphold data integrity and maintain exact participant selection throughout the study.

Signed informed consent was given by all participants and the study protocol was approved by the Ethical Committee of the Aeginition Hospital (ADA: ΩΝΚΟ46Ψ8N2-2BΨ).

### Statistics

Descriptive statistics served as the cornerstone for comparing the main demographic and clinical characteristics, providing a comprehensive snapshot of our diverse study cohort. Analysis of variance (ANOVA) was harnessed to scrutinize mean values of continuous variables, enhancing our understanding of the dataset’s variability.

Data analysis was conducted using SPSS 22. An independent *t*-test was performed to compare the mean scores on the Hamilton Anxiety Scale and between the episodic headaches group and the chronic headaches group. The *t*-test assumes normality and equal variances between groups. An independent samples *t*-test was conducted to compare depression symptom severity, as measured by the Hamilton Depression Scale, between patients with episodic and chronic headache types. The assumption of equal variances was met (*p* > 0.05).

A One-Way Between Subjects ANOVA test was conducted to examine the differences in anxiety symptom severity and depression symptom severity among patients with different headache types, e.g., episodic migraine, chronic migraine, episodic TTH, chronic TTH, episodic cluster headache (CH), new daily persistent headache (NDPH), and other types of headaches. Levene’s test confirmed the equality of variances across groups. Post hoc comparisons using the Bonferroni correction were performed to further elucidate these differences.

The statistical analysis aimed to explore the relationship between severe anxiety and depression scores and various headache characteristics, including episodic versus chronic headaches and the presence of medication overuse (MO). Binary logistic regression analysis was conducted to examine the relationship between severe anxiety scores (HAM-A ≥ 25) and the presence or absence of headaches, episodic or chronic headaches, and presence or absence of medication overuse. The logistic regression model allowed for the estimation of odds ratios (OR) and corresponding 95% confidence intervals (CI) to assess the strength and direction of the associations. In this study, we investigated the relationship between severe depression scores, categorized based on the Hamilton Depression Rating Scale (HAM-D) (cutoff > 17 indicating severe depression), and different patient groups, including those with headaches, episodic versus chronic headaches, and those with medication overuse. Logistic regression analysis was performed to examine the relationship between severe depression scores (binary outcome variable: yes/no severe depression) and patient groups (predictor variables: presence of headaches, type of headaches, medication overuse). Adjustments were made for potential confounders such as age and gender.

Logistic regression analyses were conducted to assess the associations between various symptoms (sleep disturbances, gastrointestinal symptoms, and cardiovascular symptoms) and headache disorder subtype, as well as medication overuse.

## 3. Results

The study unfolded across a substantial time frame, spanning from January 2018 to January 2021, involving a robust participant pool of 2673 individuals grappling with various forms of headache disorders (HD). Regarding the gender distribution within this cohort, the headache group consisted of 1902 women (71.2%) and 771 men (28.8%). On the other hand, 464 headache-free participants willingly accepted the opportunity to contribute to the study, resulting in participation rates of 98.3% and 91.0% for those with HD and those without, respectively. The analysis revealed migraine diagnoses in 1328 participants (49.7%), TTH in 1015 individuals (38%), CH in 139 cases (5.2%), and MO in 583 participants (21.8%). The demographic characteristics of the participants, stratified by headache disorder, are presented in Table 1, offering a clear snapshot of the diverse composition of the study cohort.

### 3.1. Scores by Headache Type

**Figure 1:**
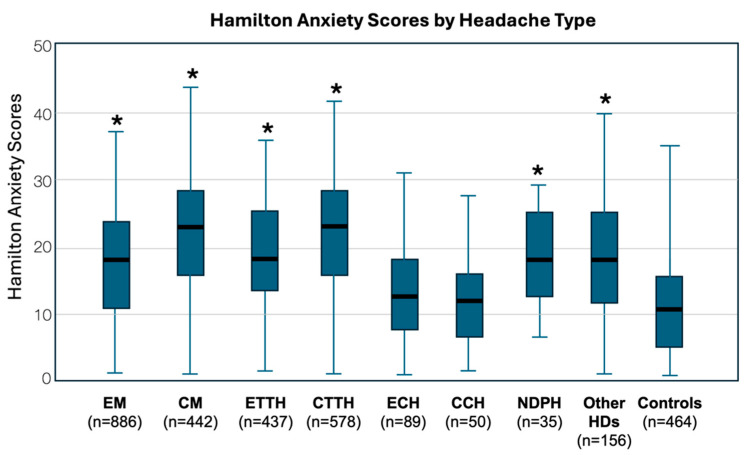
Whisker Plots of scores of Hamilton rating scales for anxiety by headache disorder. EM: Episodic Migraine; CM: Chronic Migraine; ETTH: Episodic Tension-Type Headache; CTTH: Chronic Tension-Type Headache; ECH: Episodic Cluster Headache; CCH: Chronic Cluster Headache; NDPH: New Daily Persistent Headache; HDs: Headache Disorders; Controls: Non-headache participants; * *p* < 0.001 vs. control.

**Figure 2:**
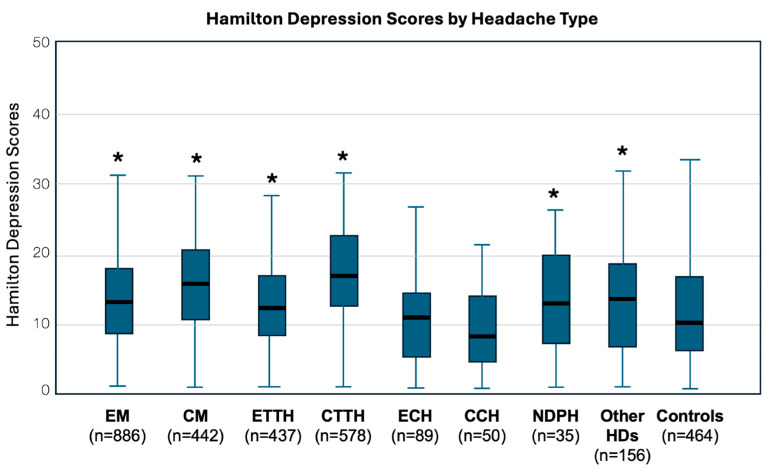
Whisker Plots of scores of Hamilton rating scales for depression by headache disorder. EM: Episodic Migraine; CM: Chronic Migraine; ETTH: Episodic Tension-Type Headache; CTTH: Chronic Tension-Type Headache; ECH: Episodic Cluster Headache; CCH: Chronic Cluster Headache; NDPH: New Daily Persistent Headache; HDs: Headache Disorders; Controls: Non-headache participants; * *p* < 0.001 vs. control.

For participants with migraine, the mean scores for HAM-A and HAM-D stood at 19.61 ± 7.69 and 14.12 ± 6.66, respectively, in contrast to participants without headaches who recorded scores of 13.09 ± 8.14 and 12.03 ± 6.96 on the corresponding scales (Table 2, Figure 1 and Figure 2). Participants with TTH reported mean HAM-A and HAM-D scores of 20.81 ± 7.54 and 15.43 ± 6.59, respectively. The mean HAM-A and HAM-D scores for participants with CH were 13.35 ± 6.69 and 10.61 ± 6.31, respectively. Participants with MO had higher scores for both HAM-A and HAM-D scales vs. participants without MO (Table 2). Furthermore, more participants with MO than participants without MO were scored ≥18 or ≥25 in HAM-A and ≥10 or ≥17 in HAM-D, indicating that more participants with MO were suffering from mild or mild to moderate or moderate to severe anxiety or mild, mild to moderate or moderate to severe depression-like condition than those without MO, respectively (Table 2).

Participants with HDs exhibited mean scores of 19.62 ± 7.79 and 14.39 ± 6.71 on the HAM-A and HAM-D, respectively. On the contrary, participants without headaches recorded lower scores of 13.09 ± 8.15 and 12.03 ± 6.96 on the respective scales.

The ANOVA revealed a significant difference in anxiety symptom severity among the various headache groups (F(7, 2665) = 47.97, *p* < 0.001). Post hoc comparisons using the Bonferroni correction were performed to further elucidate these differences. Significant differences in HAM-A severity were found between the headache groups:

- Chronic migraine patients reported significantly higher anxiety symptom severity compared to episodic migraine, episodic TTH, episodic cluster, chronic cluster, NDPH, and other types of headaches (all *p* < 0.05).
- Episodic migraine patients did not significantly differ in anxiety symptom severity from episodic TTH, NDPH, and other types of headaches (all *p* > 0.05).
- Chronic TTH patients reported significantly higher anxiety symptom severity compared to all other headache types except chronic migraine (all *p* < 0.05).
- The control group reported significantly lower anxiety symptom severity compared to patients with episodic migraine (*p* < 0.001), chronic migraine (*p* < 0.001), episodic TTH (*p* < 0.001), chronic TTH (*p* < 0.001), NDPH (*p* < 0.001), and other types of headaches (*p* < 0.001).
- There was no significant difference in anxiety symptom severity between the control group and patients with episodic or chronic CH.

A One-Way Between Subjects ANOVA test was conducted, revealing a significant difference in depression symptoms across headache types (F(7, 2665) = 23.67, *p* < 0.001). Variances were found to be equal across the groups, as confirmed by Levene’s test. Bonferroni-corrected post hoc comparisons were performed to elucidate specific differences in depression symptoms between different headache types.

- Episodic migraine participants had significantly lower depression symptom scores compared to chronic migraine patients (*p* < 0.001) and chronic TTH patients (*p* < 0.001).
- Chronic migraine participants exhibited significantly higher depression symptom scores compared to episodic migraine patients (*p* < 0.001) and episodic CH patients (*p* < 0.001). NDPH participants showed significantly higher depression symptom scores compared to chronic migraine patients (*p* = 0.011), chronic TTH patients (*p* = 0.026), and chronic CH participants (*p* = 0.023).
- Participants with other headache types demonstrated significant differences in depression symptom scores compared to chronic TTH patients (*p* < 0.001) and chronic CH participants (*p* < 0.001).
- Participants with chronic HDs (e.g., chronic migraine, chronic TTH) tend to report higher levels of depression symptoms compared to those with episodic HDs.
- Significant differences in depression symptom severity were found between various headache types, with chronic migraine and chronic TTH showing particularly elevated levels.

### 3.2. Gender, Education, Age, and Use of Anti-Depressants

Gender, education, and age were studied as confounders of the coexistence of elevated assessment for anxiety and depression in participants with headache and in the control group. Specific association for MO was additionally made.

**Figure 3:**
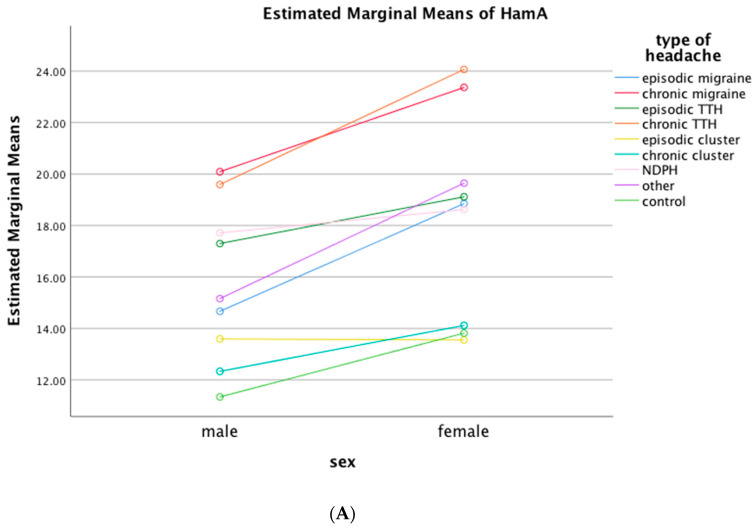

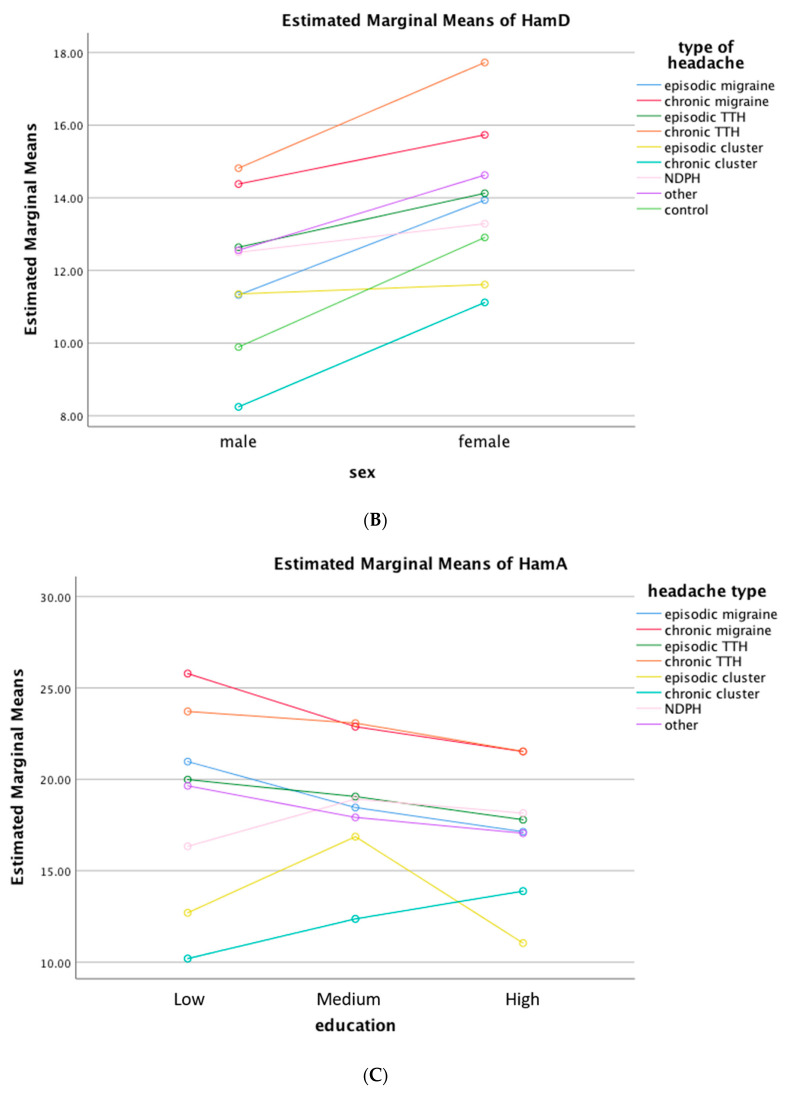

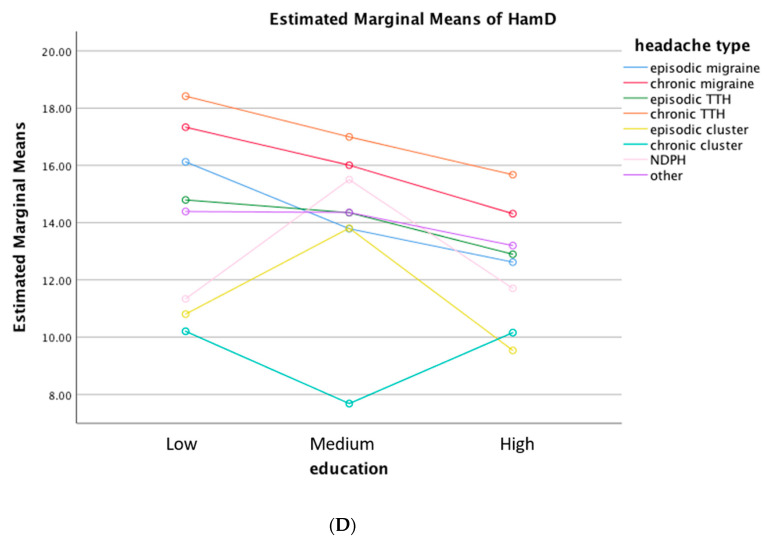
Estimated marginal means of Hamilton rating scales for Anxiety (**A**) and Depression (**B**) by headache disorder and gender or education. (**A**,**B**) Female participants had higher scores for both Hamilton rating scales in headache and non-headache participants and in all headache disorders, except for episodic cluster headache. (**C**,**D**) Except for CH and NDPH, low education level was related with high scores in both Hamilton rating scales in all HDs. HamA: Hamilton rating scales for Anxiety; HamD: Hamilton rating scales for Depression; TTH: Tension-Type Headache; NDPH: New Daily Persistent Headache; HDs: Headache Disorders; Other: participants with other Headache Disorders; Control: Non-headache participants.

- Gender influence: Females, both within headache and non-headache groups, consistently exhibited higher scores for anxiety (HAM-A) and depression (HAM-D). This trend persisted across various headache disorders, with episodic cluster headache being an exception (Figure 3).
- Education as a co-factor: The level of education was an important co-factor as well. Except for CH and NDPH, low education level was related with high scores in HAM-A and HAM-D in all HD (Figure 3).
- Impact of age: Age did not significantly contribute to the configuration of HAM-A and HAM-D scores, when analysed by headache types.
- Medication Overuse: For the case of MO, gender and education were significant co-factors. Females with MO had higher scores for both HAM-A and HAM-D than males, while low educated participants with MO also had higher scores for HAM-A and HAM-D than participants with MO and high education level. Age did not significantly affect the scoring in the context of MO.
- At the time of examination, only a small percentage of participants were under treatment with antidepressants (4.6%), while 15.5% of participants had a history of previous antidepressant treatment. Individuals with this history were more likely to score higher on both scales, but we did not find an association of prior or concurrent antidepressant use with a specific headache type among the different HD types.
- To investigate the possible impact of the COVID-19 pandemic on our study data, we performed a retrospective analysis of data collected during 2018–2019, i.e., before COVID-19, against data collected in 2020–2021. We found no significant difference, so we did not proceed with individual analyses for each headache condition.

### 3.3. Anxiety and Depression Symptoms

Symptoms of interest from the anxiety and depression scales were further examined, to explore potential associations with specific headache types. The logistic regression analyses revealed significant associations between headache types and various symptoms including sleep disturbances, gastrointestinal symptoms, and cardiovascular symptoms.

- Sleep symptoms: Sleep disturbances emerged as a very frequent feature among participants with HD, intensifying notably within the domain of chronic HD (OR = 1.252, 95% CI: 1.075–1459, *p* = 0.004) and MO (OR = 1.917, 95% CI: 1.592–2.309, *p* < 0.001) vs. participants with episodic HD and no MO, respectively.
- GI symptoms: The same pattern was observed for gastrointestinal (GI) symptoms with logistic regression analyses indicating a significant association between chronic HD and GI symptoms (OR = 1.394, 95% CI: 1.193–1.629, *p* < 0.001), as well as between MO and GI symptoms (OR = 1.510, 95% CI: 1.255–1817, *p* < 0.001).
- Cardiovascular Symptoms: While cardiovascular symptoms were notably common among participants with chronic HD, as evidenced by an odds ratio of 1.394 compared to episodic HD (95% CI: 1.193–1.629, *p* = 0.04), the influence of MO did not significantly affect the reporting of cardiovascular symptoms vs. participants without MO.

## 4. Discussion

In this cross-sectional survey encompassing 2673 individuals attending the Aeginition outpatient headache clinic, we uncovered a significant burden of severe anxiety and depressive symptoms within the headache population. Approximately 27.5% of participants with headache exhibited severe anxiety symptoms (HAM-A score ≥ 25), and 26.9% displayed severe depressive symptoms (HAM-D score ≥ 17). Notably, those grappling with chronic headache disorders (HDs) or medication overuse (MO) showcased markedly higher scores for both HAM-A (38.5% and 40.5%) and HAM-D (44.1% and 56.1%) than those with episodic HDs or no MO, respectively. Participants with migraine exhibited higher HAM-A and HAM-D scores than those with TTH or CH. Gender and education were important co-factors, but age was not. Female participants had higher scores for both HAM-A and HAM-D in headache and non-headache participants and in all HD types, except for episodic CH. Low education level was related with high scores in HAM-A and HAM-D in all HD, but not for CH and NDPH. Sleep, GI and cardiovascular symptoms were commonly reported, most often in participants with chronic than in participants with episodic HDs.

The insights garnered from a recent meta-analysis encompassing 4.19 million individuals with HDs parallel the findings of our present study. The meta-analysis identified the most prevalent comorbidities as depressive disorders (23%; 95%CI: 20–26%), hypertension (24%; 95%CI: 22–26%), and anxiety disorders (25%; 95%CI: 22–28%). These estimates closely align with the prevalence observed in our study, reinforcing the robustness of our findings. Like our study, females exhibited higher comorbidity rates, but our findings did not align with the meta-analysis regarding the association between young age and increased risk for anxiety and depression [11]. Education was not reported as a potential covariable. Why our study did not show that age is an important factor in the comorbidity of HDs with affective disorders, although the sample count was large, is not clear. It seems that other factors overshadow this relationship, possibly genetic, environmental, or even cultural. It is also highly likely that there is no such association between age and comorbidities of migraine with depression and anxiety [5,12,13]. It is noteworthy that the meta-analysis authors themselves acknowledged that their finding regarding age and comorbidity might not signify a genuine relationship. Instead, differences in the average age of subjects enrolled in the studies, particularly those above or below the age of 40, could have influenced their observations [11].

In consonance with the meta-analysis, our study underscores the significance of gender and education as co-factors in the comorbidity of anxiety and/or depression-like symptoms with HDs. Unraveling the mechanistic underpinnings of this association remains elusive, prompting the formulation of several hypothetical etiopathogenetic theories. A prevailing hypothesis, supported by a majority, suggests an interdependent relationship between anxiety, depression, and HDs. However, even within this model, discerning causation becomes intricate—whether anxiety or depression caused the headache or vice versa remains ambiguous. Instead, it appears more plausible that the coexistence of these conditions exacerbates both, demanding heightened therapeutic efforts from physicians [5,6,12,13]. Twin and family studies indicate that this bidirectional relationship can be explained, at least partly, by shared underlying genetically determined disease mechanisms. Although no genes have been robustly associated with the aetiology of both migraine and depression, genes from serotonergic, dopaminergic, and GABAergic systems together with variants in the MTHFR and BDNF genes remain strong candidates [5]. This potential genetic interplay emphasizes the intricate nature of the relationship, offering a glimpse into the complex interweaving of biological factors contributing to the co-occurrence of headache disorders and affective symptoms.

To treat these interrelated conditions, there are specific medicinal agents that have proven efficacy in the treatment of both anxiety/depressive disorders and HDs, such as the antidepressants amitriptyline and venlafaxine in the treatment of migraine [14], and amitriptyline, venlafaxine, and mirtazapine in the treatment of TTH [15]. These treatments should be the first treatment option for the specific comorbidities. Additionally, non-pharmacological therapeutic approaches, such as cognitive-behavioral treatment, physical therapy, and neurostimulation should also be considered [14,15,16], adding a multifaceted dimension to the comprehensive management of individuals navigating the intricate interplay of headache disorders and affective symptoms.

As was shown previously [3], the coexistence of depressive and anxiety like symptoms in people with HD and MO is higher compared to those without drug overuse. In other recent surveys confirming the findings of our study, individuals with MO showed a subtle psychopathological pattern characterized by impaired social adaptation [17] and depression, anxiety, and stress [18]. This nuanced understanding emphasizes the need for a thorough assessment in individuals with HDs engaged in symptomatic drug overuse. Therefore, a person with an HD who overuses symptomatic drugs for headaches should be thoroughly checked for possible coexistence of anxiety or affective disorder. Such a person, who, in addition to very frequent headaches, also has symptoms of anxiety or depression, suffers a greater deterioration in his daily quality of life and needs special care.

In our study, a special post hoc analysis was made for specific symptoms that we believe may be contributing to the chronification of primary episodic into chronic HDs. Sleep symptoms, for example, were found to be very common in participants with chronic HDs and MO, underscoring our initial hypothesis, while the same was recorded with the GI symptoms. Cardiovascular symptoms were more common in participants with chronic HD but not in participants with MO. Therefore, it appears that sleep and digestive function are disrupted much more frequently in people with chronic HD or MO, or, when an HD and anxiety or depression coexist, sleep and GI are disturbed.

Another finding that is difficult to explain is the absence of an association of cluster headache with symptoms of anxiety and depression. Cluster headache ranks as one among the most intensely painful conditions faced by man today [19], so it is reasonable to expect an increase in symptoms of anxiety and depression in those people with CH, as other studies have shown [20]. It should be noted that only a percentage of participants with CH were in an active cluster period when assessed and this may partly explain the absence of association. However, it seems that even in the case of chronic CH there is no correlation, which is troubling, because in these people the symptoms of anxiety and depression are very often intense. People with CH may often develop personality disorders, e.g., psychological dysregulation and low social engagement [20], but this cannot negate our concern regarding the absence of symptoms of anxiety and depression in this sample of people we studied. One could consider that the number of participants with CH is relatively small compared to other CH groups, and for this no significant difference was obtained, but the prevalence of CH in the general population is about 100 times lower than the prevalence of migraine and TTH, making the number of participants in the CH group in our study comparable to the number of participants in the other headache groups. In other words, the sample sizes in each HD group represent reality, so a statistically significant difference should be found, if it exists in that sample. In addition, we found only one controlled study conducted to investigate anxiety and depression symptoms in different headache types in the early 1980s. Although the classification of headaches was quite different at the time and the sample size in this survey was limited, the researchers found minimal distress in people with CH compared to people with migraine or TTH [21].

Female and low-educated participants were more likely to achieve high scores in both HAM-A and HAM-D across most, but not all, of the different headache types we investigated. In the case of CH, females did not score higher than males. One could hypothesize that most participants with CH were males in our study (104 males vs. 35 females) and this difference obscured the effect of gender, or, simply, that gender does not affect the prevalence of anxiety or depressive symptoms in people with CH, which remains low anyway. Low education was a risk factor for higher HAM-A and HMA-D scores in participants with migraine and TTH, but not in those with CH and NDPH. For the case of migraine and TTH, several studies showed an association of low education with increased burden of the conditions [15,22].

There are several methodological limitations that should be acknowledged. The survey was cross-sectional; therefore, it carries several disadvantages, e.g., it cannot be used to analyze behavior over a period of time and it does not help determine cause and effect. In addition, the timing of the snapshot is not guaranteed to be representative. Data from individuals with MO headache (MOH) are not reported, only individuals with MO, because the latter consist of individuals who although overusing medications, their headache frequency is not fulfilling the “chronic criterion” (defined as having headache for more than 15 days per month for more than three consecutive months). These individuals are at high risk of headache chronification and for MOH. Migraine with aura was not one of the conditions studied in the present study. Among the several co-factors we investigated, income is missing. Finally, the variability in headache types and the potential limitations of clustering them together for analysis should be taken under consideration.

On the other hand, the study design used in this survey offers the opportunity to compare many different variables at the same time. This inclusive approach allows for a comprehensive exploration of factors such as gender, age, and headache type in relation to the coexistence of depression and/or anxiety-like symptoms with HDs. The multifaceted analysis enriches our understanding of the complex interplay between these variables. Another advantage of this study is the relatively large sample of participants, given the fact that all they underwent was a face-to-face interview along with full neurological and physical examination to establish the diagnosis of headache. Finally, this research assessed distress in a range of different HDs and provided comparative data. There has been no other similar report over the last 40 years and none using the current headache classification.

## 5. Conclusions

In conclusion, this survey illuminates the pervasive presence of anxiety and depression-like symptoms among individuals with HDs, especially within the chronic subtypes or those grappling with MO. It is speculated that these symptoms might trigger headache attacks, acting as potential amplifiers of HD. This insight underscores the crucial role of treating physicians, emphasizing the need for comprehensive screening of individuals with headaches. Identifying and addressing anxiety and mood symptoms promptly becomes paramount, as their management holds the potential not only to alleviate these psychopathological burdens but also to mitigate the risk of headache recurrence or chronification. This proactive approach ensures a holistic and effective strategy in enhancing the overall well-being of individuals grappling with the complex interplay of headache disorders and associated mood symptoms.

## Figures and Tables

**Table 1:** Descriptive statistics.

Headache Disorder	No of Participants	Mean Age Years (SD)	Male N, (%)	Female N (%)	Primary Education	Secondary Education	Tertiary Education	Disease Duration Years (SD)
All	2.673	40.9 (12.1)	771 (28.8)	1902 (71.2)	417 (15.6%)	947 (35.4%)	1307 (48.9%)	24.2 (15.5)
Migraine	1328 (49.7%)	41.0 (12.0)	253 (19.1)	1075 (80.9)	173 (13)	495 (37.3)	659 (49.7)	24.0 (15.4)
Episodic Migraine	886 (33.2%)	41.5 (12.4)	174 (19.6)	712 (80.4)	98 (11.1)	320 (36.2)	467 (52.8)	24.5 (14.9)
Chronic Migraine	442 (16.5%)	40.0 (11.1)	79 (17.9)	363 (82.1)	75 (17)	174 (39.4)	193 (43.7)	22.9 (16.4)
TTH	1015 (38%)	41.3 (12.5)	337 (33.2)	678 (66.8)	196 (19.3)	337 (33.2)	482 (47.5)	24.8 (15.5)
Episodic TTH	437 (16.3%)	41.5 (11.9)	141 (32.3)	296 (67.7)	67 (15.3)	139 (31.8)	231 (52.9)	25.1 (15.2)
Chronic TTH	578 (21.6%)	41.2 (12.9)	196 (33.9)	382 (66.1)	129 (22.3)	198 (34.3)	251 (43.4)	24.5 (15.7)
Cluster Headache	139 (5.2%)	40.5 (11.4)	104 (74.8)	35 (25.2)	15 (10.8)	55 (39.6)	69 (49.6)	24.5 (15.5)
Episodic CH	89 (3.3%)	39.5 (11.3)	71 (79.8)	18 (20.2)	10 (11.2)	36 (40.4)	43 (48.3)	22.9 (14.9)
Chronic CH	50 (1.9%)	42.1 (11.6)	33 (66)	17 (34)	5 (10)	19 (38)	26 (52)	27.4 (16.4)
MO	583 (21.8%)	43.8 (13.1)	110 (18.9)	473 (81.1)	144 (24.7)	204 (35.0)	234 (40.1)	27.0 (14.7)
NDPH	35 (1.3%)	37.4 (8.9)	14 (40.0)	21 (60.0)	3 (8.6)	12 (34.3)	20 (57.1)	18.85 (16.2)
Other HD	156 (5.8%)	39.3 (11.7)	63 (40.4)	93 (59.6)	31 (19.9)	48 (30.8)	77 (49.4)	23.43 (15.2)
Headache Free participants	464	41.6 (14.6)	135 (29.1%)	329 (70.9%)	71 (15.3%)	165 (35.5%)	228 (49.1%)	

**Legend**. TTH: Tension-Type Headache; CH: Cluster Headache; MO: Medication Overuse; NDPH: New Daily Persistent Headache; HD: Headache Disorders; SD: Standard Deviation.

**Table 2:** Participants with anxiety or depression like conditions by headache type.

	Participants without HDs		Participants with HDs		OR	CI
	N	(%)	N	(%)		
**HAM-A ≥ 18**	173	37.3	1973	73.8	4.741	3.855–5.831
**HAM-A ≥ 25**	57	12.3	736	27.5	2.713	2.030–3.625
**HAM-D ≥ 10**	262	56.5	2006	75.0	2.319	1.892–2.842
**HAM-D ≥ 17**	115	24.8	987	36.9	1.777	1.419–2.225
	**Participants with episodic HD**		**Participants with chronic HD**			
**HAM-A ≥ 18**	889	63.0	968	76.8	1.944	1.640–2.303
**HAM-A ≥ 25**	250	17.7	486	38.5	2.915	2.441–3.481
**HAM-D ≥ 10**	1000	70.8	1006	79.8	1.625	1.359–1.944
**HAM-D ≥ 17**	431	30.5	556	44.1	1.795	1.532–2.104
	**Participants without MO**		**Participants with MO**			
**HAM-A ≥ 18**	1354	64.8	503	86.3	3.418	2.655–4.399
**HAM-A ≥ 25**	500	23.9	236	40.5	2.163	1.782–2.625
**HAM-D ≥ 10**	1491	71.3	515	88.3	3.043	2.322–3.986
**HAM-D ≥ 17**	660	31.6	327	56.1	2.768	2.294–3.339

**Legend**. MO: Medication Overuse; HD: Headache Disorder (s); HAM-A: Hamilton Rating Scale for Anxiety; HAM-D: Hamilton Rating Scale for Depression; OR: Odds Ratio; CI: Confidential Interval.

## Data Availability

Data available upon request due to restrictions. The data presented in the study are available on request from the corresponding author due to personal data protection.
